# A novel mechanism underlies atrazine toxicity in quails (*Coturnix Coturnix coturnix*): triggering ionic disorder via disruption of ATPases

**DOI:** 10.18632/oncotarget.13794

**Published:** 2016-12-04

**Authors:** Jia Lin, Hui-Xin Li, Lei Qin, Zheng-Hai Du, Jun Xia, Jin-Long Li

**Affiliations:** ^1^ College of Veterinary Medicine, Northeast Agricultural University, Harbin, People's Republic of China; ^2^ Division of Avian Infectious Diseases, State Key Laboratory of Veterinary Biotechnology, Harbin Veterinary Research Institute, Chinese Academy of Agricultural Sciences, Harbin, People's Republic of China; ^3^ Laboratory Animal Centre, Qiqihar Medical University, Qiqihar, People's Republic of China

**Keywords:** atrazine, ionic disorder, ATPases, heart and liver, Pathology Section

## Abstract

The widely used atrazine has been reported to exhibit extensive ecological hazards. Due to the biological accumulation, atrazine elicits widespread toxic effects on different organisms. However, true proof for the mechanism of atrazine-induced toxicity is lacking. To determine the potential mechanism by which atrazine exerted toxic effects, quails were treated with atrazine (0, 50, 250 and 500 mg/kg) by gavage administration for 45 days. Atrazine significantly increased the histological alterations and serum creatine kinase, lactate dehydrogenase and choline esterase levels. A marked disorder in ionic (Na^+^, K^+^, Ca^2+^ and Mg^2+^)contents and the decrease of ATPases (Na^+^-K^+^-ATPase, Ca^2+^-ATPase, Mg^2+^-ATPase and Ca^2+^-Mg^2+^-ATPase) activities were observed in the heart and liver of atrazine-exposed quails. Of note, it was also observed that atrazine suppressed the transcription of Na^+^, K^+^ transfer associated genes (Na^+^-K^+^-ATPase subunits) and Ca^2+^ transfer associated genes (Ca^2+^-ATPase subunits, solute carriers) in heart and liver. In conclusion, atrazine induced cardiac and hepatic damage via causing the ionic disorder, triggering the transcription of the ion transporters and leading the histopathological and functional alternations in the heart and liver of quails. This study demonstrated atrazine significantly induced the ionic disorder via decreasing the ATPases activities and disturbing the transcription of the ion transporters.

## INTRODUCTION

The most commonly used herbicide, atrazine, has been extensively monitored in the whole world, contaminating plants, soil, water resources [1, 2]. Atrazine, with a half-life of 30-740 days [3], is very stable in the environment due to little loss by volatilization, low sediment partitioning, and relatively slow rates of degradation [4].Of note, due to its biological accumulation effect, atrazine do a great harm to the health of organisms and human [5, 6]. Atrazine exposure at environmentally relevant doses or below resulted in endocrine disruptive effects, clear immunomodulatory, genotoxic action, metabolic disorders, neurodegenerative disorders, and so on [7-11]. Recent results from our laboratories clearly demonstrated that atrazine-induced developmental abnormality of ovary and oviduct is associated with disruption of gonadal hormone balance and hypothalamo-pituitary-ovarian axis in quails [12]. Atrazine induced hepatotoxicity and cardiotoxicity in aquatics and mammals [13-18]. Knowledge on the mechanism of these critical organs in toxicological responses to atrazine in increasing, however, has not been completely elucidated.

Numerous studies have demonstrated that exposure to atrazine induced oxidative stress, lipid peroxidation and antioxidant depletion [19-26]. In addition, it was found that atrazine exposure caused changes in erythrocytes membranes, DNA damage, mitochondrial dysfunction, cell autophagy and apoptosis [22, 25-29]. In our previous studies, atrazine is known to disturb ionic balance in mice liver and heart [18, 30], but less is known in birds.

Proper ion concentrations are benefit for a number of physiological processes such as transmembrane ionic balance, membrane potential, pH balance and cell volume to ensure correct functions of the entire body, especially the heart and liver [31]. Indeed, abnormal ion concentrations, indicating a water-electrolyte imbalance, may be partially responsible for arrhythmias, muscle contraction disorders or the incidence of death resulting from cardiovascular events [18, 32-34]. Heart and liver have been identified as the organs for atrazine-induced ionic disorders [18, 30]. However, it remains to be determined whether the effect of atrazine on the ionic imbalance contributes to organ dysfunction in birds.

Considering the central role in whole-body osmoregulation and high susceptibility to atrazine attack, ATPase is widely used as a marker for ion regulatory changes [35, 36]. In addition, ATPases are a group of membrane-bound enzymes responsible for transporting the cations across the cell membrane, maintaining intracellular functions [37, 38]. Although it is reported the effects of atrazine on ionic disorder and ATPases disturbance in mice, the data concerning the birds were limited. Therefore, the aim of our study was to investigate the deleterious effects of atrazine on the heart and liver of quails, the involvement of the ionic disorder, and the possible mechanism of atrazine-induced organs damage.

## RESULTS

### Effects of atrazine on heart/liver weight and biochemical parameters analyses in quails

**Figure 1:**
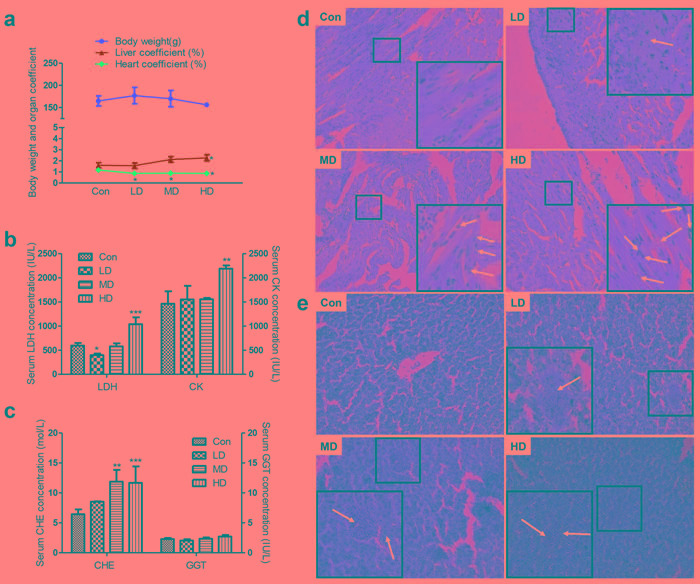
Effects of atrazine on the organ coefficient, biochemical analysis and histopathological in quail heart and liver **a.** Body weight and organ coefficient; **b.** The LDH and CK activity in serum; **c.** The CHE and GGT activity in serum; **d.** The histopathological analysis in heart; **e.** The histopathological analysis in liver. The magnification of the microscope is 40×10 times the original size, and the arrows in white point to the location of the lesion. Values were expressed as mean ± S.D.. Symbol for the significance of differences between the vehicle control and another: * *P* < 0.05, ** *P* < 0.01, *** *P* < 0.001.

The animals in groups all remained relatively in good health throughout the study and there were no exposure-related clinical observations. Furthermore, atrazine had no effect on feed intake or body weights in male quails. Interestingly, we found that the heart coefficient decreased (*P* < 0.05) in quails of HD group and the liver coefficient increased (*P* < 0.05, *P*< 0.05, *P*< 0.05) in all atrazine-exposed quails, respectively (Figure 1a).

### Effects of atrazine on the biochemical and histopathological analyses

Regarding the biochemical analyses, the serum LDH was significantly pronounced upon decrease by atrazine at LD group (*P* < 0.05; Figure 1b). We also checked that serum LDH and CK levels were increased after treat with atrazine at HD group (*p* < 0.001, *p* <0.01; Figure 1b), as LDH and CK had been suggested to impact the function of heart. We then detected CHE and GGT in serum. No difference in serum GGT was observed between Con and atrazine-exposed groups (Figure 1c). Additionally, serum CHE was found to increasingly evident with dosage of atrazine (Figure 1c), especially in MD and HD groups (*P* < 0.01, *P* < 0.001), suggesting an injury in atrazine-exposed quail liver.

In order to investigate the effect of atrazine in organ injury, we conducted histological analysis of heart and liver sections, which measured the degree of organ injury. Cardiomyocytes of atrazine-exposed quails exhibit an increase in the number of nuclear vacuoles, especially marked promotion of nuclear vacuoles in MD and HD groups (Figure 1d). Myocardial fiber dissolution or fracture can be found in HD group (Figure 1d). Swelling of cardiac myocytes was observed in LD group (Figure 1d). Analyzing microanatomy alterations in hepatocytes, we found hyperaemia in central vein and hepatic sinusoid with dose-related increases (Figure 1e). The hepatocyte swollen became more serious as the dose of atrazine increases (Figure 1e). In addition, the derangement of hepatic plate aggravated gradually with the dosage increasing (Figure 1e) was observed in atrazine-exposed quails.

### Detection of Na^+^, K^+^ content, Na^+^-K^+^-ATPase activity and ATPase subunit transcription

**Figure 2:**
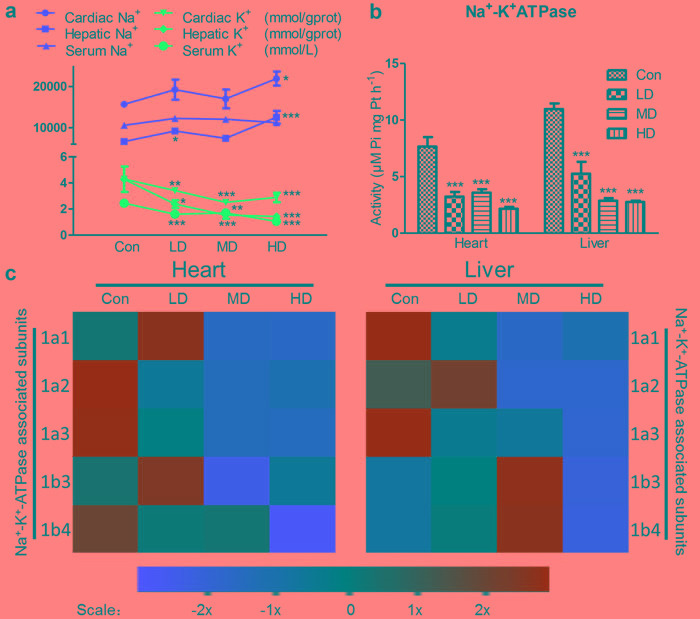
Effects of atrazine on the modulation of K^+^ transfer channel **a.** The Na^+^, K^+^ content in heart, liver and serum; **b.** The Na^+^-K^+^-ATPase activity in heart and liver; **c.** The heat-map of Na^+^-K^+^-ATPase associated subunits in heart and liver. Values were expressed as mean ± S.D.. Symbol for the significance of differences between the vehicle control and another: * *P* < 0.05, ** *P* < 0.01, *** *P* < 0.001. The mRNA expression levels of genes transcription are shown using the indicated pseudo color scale from -2x (green) to +2x (red) relative to values for Con group. The color scale represents the relative mRNA expression levels, with green indicating up-regulated genes, red indicating down-regulated genes, and black indicating unchanged genes.

To investigate the effects of Na^+^-K^+^-ATPase on Na^+^, K^+^ disorder directly in heart and liver, we tested Na^+^, K^+^ content, Na^+^-K^+^-ATPase activity and ATPase subunit transcription. K^+^ content was deeply suppressed by atrazine in the heart, liver and serum, as compared with Con group (Figure 2a). Na^+^ content was significantly increased in the heart and liver in HD group (*p* < 0.01, *p* < 0.001), the serum Na^+^ didn't show any difference in groups (Figure 2a). Similar to function of serum Na^+^, serum Cl^-^ help to maintain the electrolyte homeostasis, acid-base equilibrium and osmotic balance. However, we did not observe changes in serum Cl^-^ (Figure S1). The activities of cardiac and hepatic Na^+^-K^+^-ATPase in the atrazine-treated groups were shown in Figure 2b. The Na^+^-K^+^-ATPase activities in the atrazine-treated groups significantly decreased (*P* < 0.001) in all atrazine treated groups compared to Con group on heart and liver. The maximal adverse effects were observed for cardiac and hepatic Na^+^-K^+^-ATPase activities after the administration of 500 mg/kg atrazine (*P* < 0.01).

Since transcription of Na^+^-K^+^-ATPase can alter the activity of Na^+^-K^+^-ATPase, we next detected the expression of Na^+^-K^+^-ATPase associated subunits transcription. These results revealed a set of Na^+^-K^+^-ATPase subunits (1a1, 1b3) in quail heart whose transcription was first increased in LD and then decreased in MD and HD significantly by atrazine treatment, compared to Con group. Additionally, a set of Na^+^-K^+^-ATPase subunits (1a2, 1a3, 1b4) genes was decreased by atrazine in heart, compared to Con group (*P* < 0.001) (Figure 2c; Figure S2a). Inspection of the results of atrazine-induced genes transcription alterations in liver and heart appeared to have a similar, albeit more variable, effect. In the liver, a set of Na^+^-K^+^-ATPase subunits (1a2, 1b3) was first increased in LD and then decreased markedly in HD. A set of Na^+^-K^+^-ATPase subunits (1a2, 1a3) in quail liver was decreased induced by atrazine treatment (Figure 2c; Figure S2b). The effects of atrazine-induced Na^+^-K^+^-ATPase activity down-regulating were likely due to lower expression levels of the transcription of Na^+^-K^+^-ATPase associated subunits (Figure 2b, 2c; Figure S2).

### Detection of Ca^2+^ content, Ca^2+^-ATPase activity and Ca^2+^ transport associated subunits transcription

**Figure 3:**
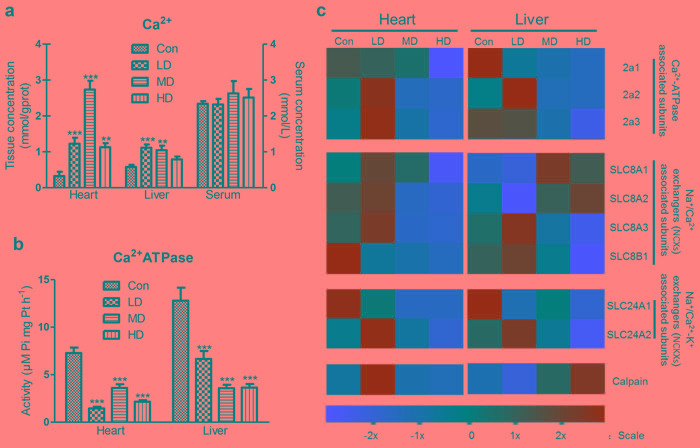
Effects of atrazine on the modulation of Ca^2+^ transfer channel **a.** The Ca^2+^ content in heart, liver and serum; **b.** The Ca^2+^-ATPase activity in heart and liver; **c.** The heat-map of Ca^2+^-ATPase associated subunits, calpain and SLCs. Values were expressed as mean ± S.D.. Symbol for the significance of differences between the vehicle control and another: * *P* < 0.05, ** *P* < 0.01, *** *P* < 0.001. The mRNA expression levels of genes transcription are shown using the indicated pseudo color scale from -2x (green) to +2x (red) relative to values for Con group. The color scale represents the relative mRNA expression levels, with green indicating up-regulated genes, red indicating down-regulated genes, and black indicating unchanged genes.

To address the role of Ca^2+^ disorder in organ injury, we tested not only Ca^2+^ content, Ca^2+^-ATPase activity and ATPase subunits transcription, but also calpain, the Na^+^/Ca^2+^ exchangers (NCXs) and the Na^+^/Ca^2+^-K^+^ exchangers (NCKXs) associated subunits gene transcription in heart and liver. The cardiac Ca^2+^ content was significantly increased in LD, MD and HD groups (*P* < 0.001, *P* < 0.001, *P* < 0.005). Similar to alterations in heart, the hepatic Ca^2+^ content was significantly increased in LD and MD groups (*P* < 0.001, *P* < 0.005). However, we also observed that there were no significant alterations in serum Ca^2+^ of atrazine-exposed quails (Figure 3a). Moreover, the activities of Ca^2+^-ATPase in heart and liver of atrazine-treated quails were significantly decreased (*P* < 0.001) in all atrazine treated groups (Figure 3b).

A heat map showed the expressions of the 3 Ca^2+^-ATPase subunits, calpain and solute carriers (SLCs) in atrazine-treated quails (Figure 3c). These results revealed that the expression of Ca^2+^-ATPase subunit 2a1 in heart decreased in MD and HD groups (Figure 3c), compared to Con group (*P* < 0.01, *P* < 0.001) (Figure S3a). Another set of genes (2a2, 2a3) in heart was first increased in LD group and then decreased in HD group (Figure 3c), compared to Con group (*P* < 0.05) (Figure S3a). Similar to the alterations in heart, a set of Ca^2+^-ATPase subunits (2a1, 2a3) expression was markedly decreased in MD and HD groups (Figure 3c), compared to Con group (*P* < 0.001, *P* < 0.001) (Figure S3b). The expression of Ca^2+^-ATPase subunit 2a2 in quail liver was first increased in LD and then decreased in HD group (Figure 3c), compared to Con group (*P* < 0.001, *P* < 0.05) (Figure S3b). Moreover, we observed that expression of calpain in heart was significantly increased in LD group (*P* < 0.001) (Figure 3c; Figure S3c). The hepatic calpain transcription was significantly increased in MD and HD groups (*P* < 0.01, *P* < 0.001; Figure 3c; Figure S3d).

The NCXs form the SLC8 family, with NCX1, NCX2 and NCX3 being coded for by the genes SLC8A1, SLC8A2 and SLC8A3 respectively [39, 40]. The NCKXs form the SLC24 family, with the five members being coded for by the genes SLC24A1- SLC24A5 and some splice variants exist [41]. The expressions of the SLCs (SLC8A1-3, SLC8B1, SLC24A1-2) in liver and heart were tested in this experiment. Almost all of the SLCs in heart were significantly suppressed in HD group (Figure S3e). However, SLC8A1 and SLC8A2 in liver were significantly enhanced in MD and HD groups (Figure S3f), and SLC8A3, SLC8B1, SLC24A1, SLC24A2 were significantly decreased in HD group (Figure S3f).

### Detection of Mg^2+^ content and the activities of Mg^2+^-ATPase and Ca^2+^-Mg^2+^-ATPase

**Figure 4:**
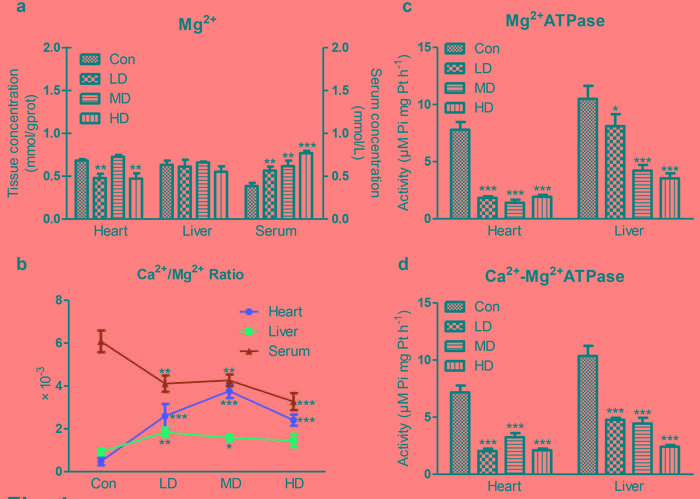
Effects of atrazine on the modulation of Mg^**2+**^ transfer channel **a.** The Mg^2+^ content in heart, liver and serum; **b.** The Ca^2+^/Mg^2+^ ratio in quail heart, liver and serum; **c.** The Mg^2+^-ATPase activity in heart and liver; **d.** The Ca^2+^-Mg^2+^-ATPase activity in heart and liver. Values were expressed as mean ± S.D.. Symbol for the significance of differences between the vehicle control and another: * *P* < 0.05, ** *P* < 0.01, *** *P* < 0.001.

Cardiac Mg^2+^ content showed a significant decrease in LD and HD groups compared to Con group (*P*< 0.01; Figure 4a). However, hepatic Mg^2+^ content showed no significant alteration in atrazine-exposed groups compared to Con group (Figure 4a). The serum Mg^2+^ content showed a significant increase in atrazine-exposed groups compared to Con group (*P* < 0.01, *P* < 0.01, *P* < 0.001; Figure 4a). The Ca^2+^/Mg^2+^ ratio was significant increase in heart at all atrazine treated groups (*P* < 0.001; Figure 4b) and in liverat LD and MD groups (*P* < 0.01, *P* < 0.05; Figure 4b); however, the Ca^2+^/Mg^2+^ ratio was significant decrease in serum (*P* < 0.01, *P* < 0.01, *P* < 0.001; Figure 4b).

Figure 4c showed a significant decrease in cardiac Mg^2+^-ATPase activity in atrazine-exposed groups compared to Con group (*P* < 0.001, *P* < 0.001, *P* < 0.001). Similar to the alterations in heart, a significant decrease in hepatic Mg^2+^-ATPase activity in atrazine-exposed groups (*P* < 0.05, *P* < 0.001, *P* < 0.001) was also observed. The results of Ca^2+^-Mg^2+^-ATPase activity after atrazine exposure were presented in Figure 4d. There was a significant decrease on Ca^2+^-Mg^2+^-ATPase activity of atrazine-exposed groups compared to Con group (*P* < 0.001, *P* < 0.001, *P* < 0.001) in heart and liver. These data, in light of previous reports including ours [18, 30] stating that atrazine-induced Mg^2+^ disorder was increased by disturbing the activities of Mg^2+^-ATPase and Ca^2+^-Mg^2+^-ATPase.

### PCA of cardiac and hepatic ionic homeostatic modulation

**Figure 5:**
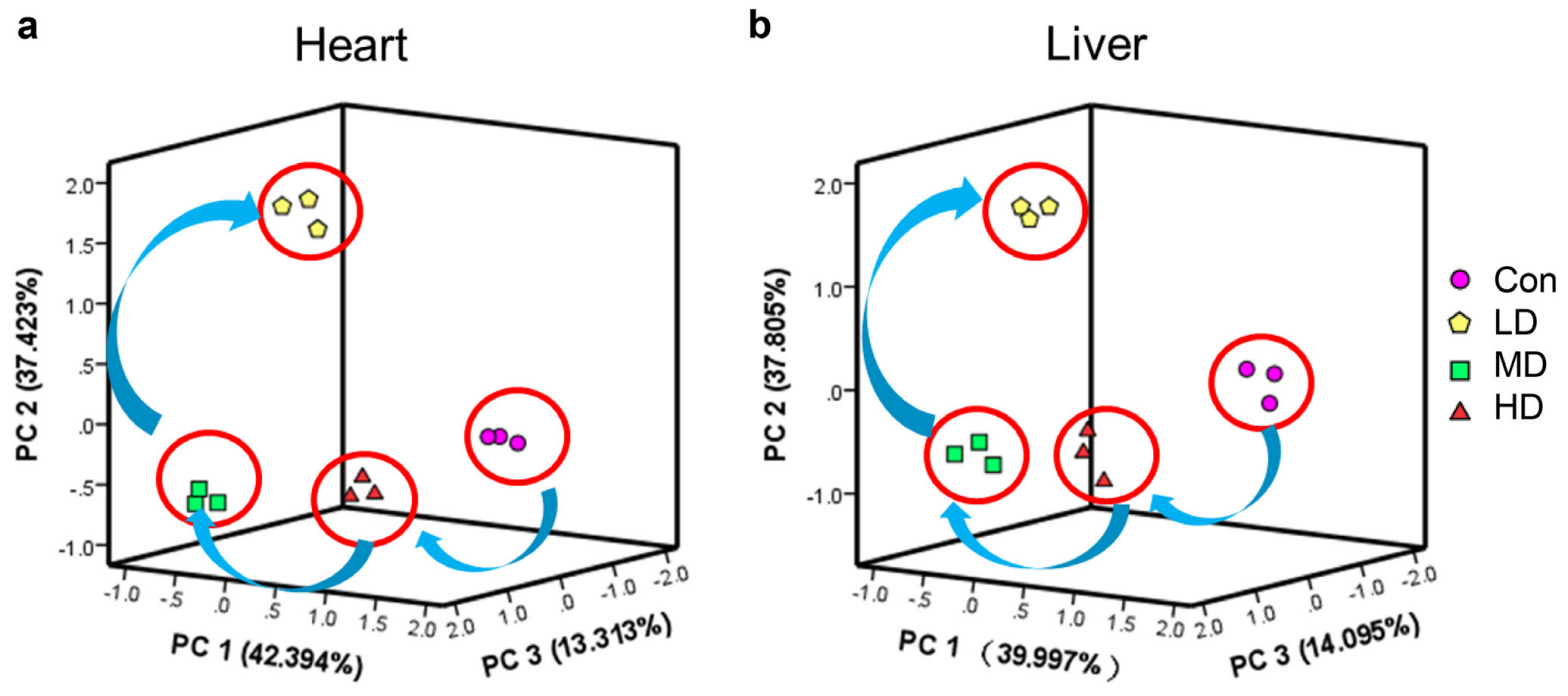
PCA of ionic homeostatic regulation after treated with atrazine PCA score plot results comparing biochemical parameters of 4 treatment groups. **a.** PCA of ionic homeostatic regulation in heart; **b.** PCA of ionic homeostatic regulation in liver.

PCA for the relevant metabolites was performed with an analysis of variance (*P* < 0.05), and the results were depicted in Figure 5. PC1, PC2 and PC3 conglomerated more than 80% of the total variance (Table S2). Cross-validation allowed checking of the predictive power of the generated model using the Q2/R2 ratio as a measure of the reliability of the predictions. This ratio was above 0.8 for PC1, PC2 and PC3, suggesting that the generated model was consistent and valid (Table S2).

To distinguish potential effects of biochemical parameters in quails receiving different doses of atrazine, PCA was performed as an unsupervised pattern recognition method. The PCA scores showed that the dose-related separation within the atrazine-treated animals was quite remarkable in both heart and liver, and the changes in metabolic profiles from the controls to the low- middle- and high-dose groups occurred in a clockwise direction; while clearly separated from the controls (Figure 5). Taken together, these results showed that atrazine exposure resulted in dose-related changes in biochemical parameters in both heart and liver.

## DISCUSSION

Atrazine can interfere with the normal operation of the cardiovascular system leading to a broad range of disorders, increased rates of cardiovascular diseases [18, 42]. Liver is viewed as a primary organ for atrazine metabolism, which is known to cause hepatic damage in mammalian [15, 43, 44]. However, whether the effects of atrazine have the potential influence to the cardiac and hepatic function of birds still remain unclear. The quail is increasingly used as a model species for studies of stress physiology including the effects of the exposure to environmental chemicals such as atrazine. Of note, our findings are in accordance with previous studies [18, 30] that showed atrazine induced cardiotoxicity and hepatotoxicity in mice via the disturbance of ionic balance, but molecular disease mechanisms remain to be elucidated in birds.

Atrazine had been reported to interfere the growth and development of quails, to disturb its reproductive function, to induce endocrine disruption and to trigger the pathological and genotoxic effects [7, 11, 45]. In the present study, nomortality occurred at any dose of ATR administered. There were no consistent effects of ATR on body weight and clinical behavior alterations on female [12] and male quails, which might be attributable to the effects of ATR on sexual organs damage in different pathways.

In this study, significantly ionic disorders and organs injury were observed in the liver and heart of quails. According to previous studies, atrazine can induce pathological effects in multiple tissues [7, 12]. Atrazine was recorded in the liver and heart [46-49], suggesting the heart and liver are organs involved in the metabolism of atrazine. Our previous study showed that atrazine induced significantly ionic disorder in the liver and heart on mice as well [18, 30]. Therefore, the liver and heart have been identified as the organs for atrazine-induced ionic disorders. In accordance with our findings in quails, atrazine stimulated ionic homeostasis disturbance in aquatic and mammalian [18, 30, 50]. Since ions are involved in a number of physiological processes, proper ion concentrations are crucial to maintain regular function of the heart and liver. Atrazine led to heart, liver and serum ionic disorders, induced structural alterations and dysfunction on heart and liver in quails. ATPases, which constitute a major category of ion transporters, are significantly suppressed by atrazine in aquatics and mammals [18, 30, 51, 52]. Our results showed the similar effects on ATPases activities in quails after atrazine exposure. In addition, SLCs gene levels were significantly decreased in heart and partially decreased (except SLC8A1 and SLC8A2) in liver. These results demonstrated that atrazine can induce ionic disorders by altering ATPases and contribute to organ dysfunction during intoxication.

**Figure 6:**
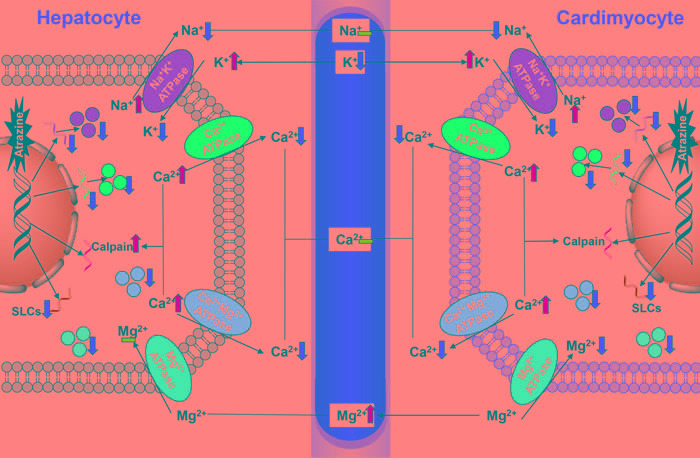
The pathway of atrazine induced ionic disorder in the heart, liver and serum

There are many ionic pumps and ion channels involved in Na^+^, K^+^ homeostasis. Among them the most important one in all living cells is Na^+^-K^+^-ATPase, which actively transport K^+^ into and Na^+^ out of the cells, responsible for the balance between Na^+^, K^+^ intra- and extracellular concentration, maintaining Na^+^, K^+^ serum levels [32]. Numerous studies determinated that atrazine-induced Na^+^, K^+^ disorders are associated with the inhibition of Na^+^-K^+^-ATPase [18, 30, 51, 53, 54]. In the current study, atrazine decreased significantly the concentrations of cardiac, hepatic K^+^, which revealed that the decrease of cardiac and hepatic K^+^ may be a response to the increase of K^+^ outside the cardiomyocytes and hepatocytes via disturbing Na^+^-K^+^-ATPase on quails. The changes of contents of K^+^ of experimental group in the liver were more obvious than heart's, suggesting that atrazine could disorder K^+^ content directly via influencing Na^+^-K^+^-ATPase activities. Moreover, atrazine caused cardiac, hepatic, nephric dysfunction, activated renin-angiotensin-aldosterone system, boosted levels of aldosterone [55]. The role of aldosterone is to promote K^+^ excretion and Na^+^ reabsorption. Thus, the contents of K^+^ in serum drop as well. The decreased Na^+^-K^+^-ATPase activity causes retention of Na^+^ in the cells. These results suggested that atrazine delivered cardiotoxicity and hepatotoxicity in quail via the decreased of Na^+^-K^+^-ATPase activity and subunit transcription as well as disorders in K^+^ levels (Figure 6).

Changes in Ca^2+^ are very important during signal transduction processes involved in cellular functions [56, 57]. In cardiac myocytes, elevated cytosolic free Ca^2+^ required for muscle contraction should be removed rapidly to ensure relaxation; otherwise the overloading of Ca^2+^ activated a variety of Ca^2+^-dependent degradation enzymes, inducing arrhythmia and myocardial damage. In hepatocytes, CD38-mediated Ca^2+^ signaling contributes to angiotensin II-induced activation of hepatic stellate cells, inducing hepatic fibrosis [58]. Additionally, Ca^2+^ over loading stimulate mitochondrial damage, increase free radicals; free radical damage to the membrane, increased Ca^2+^ permeability, and then in turn promote Ca^2+^ overloading, induce tissues injury. Atrazine is considered to be undesirable because of its adverse effects on living organisms to induce Ca^2+^ release [18, 30, 59, 60]. Atrazine exposure was accompanied by significant decrease in Ca^2+^-ATPases and disturbed Ca^2+^ homeostasis [59]. The overall increase in Ca^2+^ concentration in heart and liver of quails after atrazine exposure may disturb organ function in this study.

NCX, as a bidirectional antiporter, is membrane transporters [61-63]; the ion-exchange process is electrogenic, with a stoichiometry of 3Na^+^ for 1Ca^2+^ [64]. NCKX, a K^+^-dependent Na^+^/Ca^2+^ exchanger, exchange 4Na^+^ for 1Ca^2+^ and 1K^+^ [39]. Na^+^/Ca^2+^ exchangers (NCX, NCKX) use the Na^+^ electrochemical gradient across the plasma membrane to extrude intracellular Ca^2+^ and maintain Ca^2+^ homeostasis. NCXs and NCKXs are encoded by SLCs [61, 65-68]. In the present study, we also found that SLCs and Ca^2+^-ATPase associated subunits transcriptions were significantly decreased by atrazine, which may be responsible for the overloading of Ca^2+^. Moreover, the retention of Na^+^ in the cells leads to the inhibition of the NCXs expression and the Ca^2+^ overload in the cells. The further overloading of Ca^2+^, the increase of Na^+^ by Na^+^-K^+^-ATPase were relieved by NCX. Thus, the changes of Na^+^ contents were not obvious compared with the K^+^ contents. As a consequence of Ca^2+^ influx by atrazine, the transcription of calpain is partially activated. Although the change of the activity of Ca^2+^-ATPase was extremely obvious in liver, the changes of the NCXs expression were more extremely obvious in heart. Additionally, Unlike Na^+^ channels, Ca^2+^ channels are also regulated by neurotransmitters. Several studies had reported that atrazine could activate cAMP [69-71] and then activate L-type Ca^2+^ channel, inducing the influx of Ca^2+^. However, the excretion of Ca^2+^ is inhibited by Ca^2+^-ATPase. The overloading of Ca^2+^ in the hepatocytes can be discharged to the bile by the activation of cAMP. Thus, the changes of the Ca^2+^ content were not obvious in liver compared with in heart. These findings emphasize the view that the decreased Ca^2+^-ATPase activity may contribute to the disturb of intracellular Ca^2+^ homeostasis by increasing Ca^2+^-ATPase associated subunits and SLCs mRNA levels, leading the organ dysfunction (Figure 6).

Atrazine-induced malfunction in tissues is associated with Mg^2+^ imbalance [72, 73]. Mg^2+^ is a cofactor for several enzymes such as Na^+^-K^+^-ATPase and Ca^2+^-ATPase that maintain the ionic balance [74]. Both pumps are Mg^2+^ dependent, and Mg^2+^ deficiency can impair ATPases functions [75, 76]. Conversely, Mg^2+^ deficiency leading to damage to ATPases [77]. Additionally, the recent research raise the issue of Mg^2+^ is a physiologically important regulator of Ca^2+^ channel function [78]. In the present study, atrazine exposure caused increase of the Ca^2+^/Mg^2+^ ratio thus eliciting ionic imbalance, inducing the ATPases disorder, and disrupting the function of heart and liver. Moreover, atrazine disturbed the Mg^2+^ balance and organ function on quails via decreasing the Mg^2+^-ATPase and Ca^2+^-Mg^2+^-ATPase.

In conclusion, atrazine induced cardiac and hepatic damage via causing the ionic disorder, triggering the transcription of the ion transporters and leading the histopathological and functional alternations in the heart and liver of quails. However, our study demonstrated atrazine significantly induced the ionic disorder in the organ via modulating the ATPases activities and disturbing the transcription of the ion transporters (Figure 6). Therefore, it is hypothesized that triggering ionic disorder and disruption of ATPases is the novel mechanism of atrazine-induced toxicity. This study provides novel insights into the ionic disorder and toxicological responses to atrazine.

## MATERIALS AND METHODS

### Animals and treatments

Male quails (Coturnix C. coturnix) aged 18 days and weighted 89.04 ± 3.66 g were purchased from Wan Jia farm in Harbin, China. Chemical atrazine (C_8_H_14_ClN_5_, CAS: 1912-24-9) was purchased from Zhonghe Chemical Limited Company (Binzhou, China). Birds were housed in cages in an environmentally controlled room (temperature 26 ± 2□ and humidity 50 ± 15%). Temperature and relative humidity were monitored daily. Feed and water were offered *ad libitum* during the experiment. After one week acclimation, the quails were randomly divided into four groups (Table 1). Birds were administered atrazine once a day orally by gavage for 45 days. The gavage concentration of atrazine was 0, 50, 250, 500 mg/kg bodyweight for each group. Usually effects appear late after exposure due to the time needed to modify the compensation mechanisms that maintain the homeostasis of organisms. To cover these long-term effects, possible alterations in the experimental animals were monitored at 45 days after the beginning of treatment. All of this makes the conditions used in the present work closer to realistic field situations [45].

The current ecological risk assessment for atrazine in avian species established by the USEPA reports a dietary LOAEL (lowest observable adverse effect level) of 675 mg/kg in the northern bobwhite quail. In this study, all the experiments conducted in animals were in accordance with the guidance of ethical committee for research on laboratory animals.

At the end of the experiment, the quails were weighed. Then, the birds were fasted before the day of sacrifice, and their heart and liver were carefully dissected out and weighed, then storing at -80 □ for assays. And the organ coefficient is obtained by the ratio of organ weight to body weight. The blood was collected from the heart of each bird and centrifuged at 3000 rpm for 10 min to obtain the serum. The serum were stored at -80 □ for assays.

**Table 1:** Animal groups

Groups	Number	Drug	Concentration
Con (Control)	50	Pure water	0
LD (Low-dose atrazine)	50	Atrazine	50 mg/kg
MD (Middle-dose atrazine)	50	Atrazine	250 mg/kg
HD (High-dose atrazine)	50	Atrazine	500 mg/kg

### Determination of biochemical parameters

Blood samples were used to investigate changes in the serum enzymes and concentration of ions considered to be biochemical indicators of hepatic and myocardial enzyme. Serum Na^+^, K^+^, Ca^2+^, Mg^2+^, Cl^-^ concentrations and the activities of creatine kinase (CK), lactate dehydrogenase (LDH), choline esterase (CHE), glutamyltranspeptidase (GGT) were measured. The activities or contents of biochemical parameters were detected using the detection kits (Jiangsu SINNOWA Medical Technology Company, China) by a biochemical auto-analyzer.

### Histological and ultrastructural observations

Cardiac and hepatic specimens were fixed in 10% buffered neutral formalin and were processed for paraffin wax sectioning. Sections of about 5 μm thickness were stained with hematoxylin and eosin for light microscopy.

### ATPase activity assays

The activities of Na^+^-K^+^-ATPase, Ca^2+^-ATPase, Mg^2+^-ATPase and Ca^2+^-Mg^2+^-ATPase were determined using the appropriate assay kits (Nanjing Jiancheng Bioengineering Institute, China) according to the manufacturer's instructions using 10% tissue homogenates [18, 30].

### RNA purification and Quantitative real-time PCR

Total mRNA was extracted from hypothalamo, pituitary and ovary using RNAout reagent (Beijing Tiandz, Inc. China), according to the manufacturer's instructions. First cDNA strand was synthesized using TransScript All-in-One First-Strand cDNA Synthesis SuperMix for quantitative real-time PCR (qRT-PCR) (One-Step gDNA Removal) (Beijing TransGen Biotech Co. Ltd., China). The primers for real-time amplification of relative cDNAs were designed using Oligo 7.22 Software (Molecular Biology Insights, Cascade, CO) based on the deposited sequences in GenBank and primers used are given in Table S1 of the Supplementary Material. qRT-PCR was conducted using LightCycler® 480 Real-Time PCR System (Roche, CH). Triplicate samples were assessed for each gene of interest, and β-actin was used as a control gene. Relative expression levels were determined by the 2^-ΔΔCt^ method [79], the results were normalized to the mean of ACTB.

### Statistical analysis

The data was analyzed with GraphPad Prism 5.1 (GraphPad Software Inc., USA) and SPSS 19.0 software (SPSS Inc., USA). Statistical analyses were performed using one-way ANOVA followed by Tukey's post hoc pairwise comparison. Asterisks (*) indicate statistically significant differences from the control group, *P < 0.05, **P < 0.01 and ***P < 0.001. Ranking of genes by degree of differential expression was analyzed with a heat map using the R Programming Language version 3.2.1. In addition, Principal component analysis (PCA) was used as an effective tool for simplifying the information from inter-correlated variables through linear transformation of the original variables into a few principal components. PCA was performed in this work to define the most important parameters, which could be used as key factors for individual variations using the same software. The observed relationships among the parameters were confirmed and quantified according to a Spearman's test. All data was presented as mean ± standard deviation (SD). In addition, Chem Draw Pro (version 15.0) was used for drawing pictures.

## SUPPLEMENTARY MATERIALS TABLES AND FIGURES


