# Supplementary material for: A novel mechanism underlies atrazine toxicity in quails (Coturnix Coturnix coturnix): triggering ionic disorder via disruption of ATPases
Source: Oncotarget. 2016 Dec 4;7(51):83880–92. doi: 10.18632/oncotarget.13794 (PMC5356632; doi:10.18632/oncotarget.13794)
Supplement: Supplementary file 1 [file oncotarget-07-83880-s001.pdf]

A novel mechanism underlies atrazine toxicity in quails (*Coturnix Coturnix coturnix*): triggering ionic disorder via disruption of ATPases

Supplementary Materials

Table S1. Oligonucleotides used in the present study.

	Gene	Forward Primer（5’→3’）	Reverse Primer（5’→3’）	Product length	NCBI accession No.
**House keeper**	**ATCB**	CCGTGTTCCCATCCATCGTG	GCCAGATCTTCTCCATATCATCCC	174 bp	NM_205518.1
		GTGACCTGACGGACTACCTCA	TCTCCTGCTCGAAATCCAGT	132 bp	
**$Na^{+}K^{+}$ ATPase associated subunits**	**1a1**	TACAGCCTTCTTTGTCAGCA	CAGCTAGAGCAGTCTCCTCGAA	140 bp	NM_205521.1
	**1a2**	GCCGCTGTCGTCATTGTCA	TCTCCGCGTTGATTTGGAT	142 bp	NM_205476.1
	**1a3**	CCATCCTCAAGCGTGACGTG	ACTTTGACGGACCCGGACGA	85 bp	NM_205475.1
	**1b3**	GCAGTTCGTCTACAACCCCA	TAAGCCCTGGACTAGAAATCCG	194 bp	NM_205535.1
	**1b4**	GCCAGGAGTAATGATCAGACCA	TCATCGTAAGCTGCTAGGAAC	120 bp	NM_001044651.1
**Ca^{2+} ATPase associated subunits**	**2a1**	TCACCACCAACCAGATGTCC	GAGAAGAATCCGCACAAGCAG	145 bp	NM_205519.1
	**2a2**	GCGATTTGCTTGTCCATGTCAC	ATCAGCCATTGCGTCACGTT	101 bp	NM_001271973.1
	**2a3**	ACATCCGCATCATTGAAATCCG	ACCCCTGTTGCAATGACGAT	199 bp	NM_204891.1
**Sodium calcium exchanger (NCX)**	**SLC8A1**	TCTACAAGAAATACCGAGCTG	AATTAGCAACCTTTCCGTCCA	108 bp	NM_001079473.1
	**SLC 8A2**	TTCGAGCCCTGCCTCTACCAC	GCCGTCCTCCGTCTTGTAGTCCA	120 bp	XM_015272480.1
	**SLC 8A3**	TAGAGGTCATTACATCGCAAG	ATTCCAAATCCGAATGGTTGT	86 bp	NM_001293097.1
	**SLC8B1**	AAGAGCCACCCAAATACCACT	GCTGCATTGATCCACATAGCG	91 bp	XM_004934497.2

	**SLC24A1**	CAGCACCCAAAATAAGGCACT	GCCAAAGATGTGAAGTACAACCC	206 bp	NM_001001773.1
	**SLC24A2**	CTGCTGGCACATCCATTCCT	TGCACAGAACAGACCATTGCT	197 bp	NM_001001772.1
	**Calpain**	AGAACATCATCCCAGCGT	AGCCTTCACTACCCTCTTG	229 bp	NM_205303.1

Table S2. Total Variance Explained of PCA. (a) Total Variance Explained of PCA for Figure 5a in heart; (b) Total Variance Explained of PCA for Figure 5b in liver.

Table S2a. Total Variance Explained of PCA for Figure 5a in heart.

Total Variance Explained

Component	Initial Eigenvalues			Extraction Sums of Squared Loadings			Rotation Sums of Squared Loadings		
	Total	% of Variance	Total	Total	% of Variance	Total	Total	% of Variance	Total
1	11.603	50.450	50.450	11.603	50.450	50.450	9.751	42.394	42.394
2	7.044	30.625	81.075	7.044	30.625	81.075	8.607	37.423	79.817
3	2.773	12.055	93.130	2.773	12.055	93.130	3.062	13.313	93.130
4	.934	4.062	97.193						
5	.262	1.139	98.331						
6	.189	.820	99.151						
7	.095	.413	99.565						
8	.051	.221	99.785						
9	.027	.116	99.901						
10	.014	.061	99.963						
11	.009	.037	100.000						
12	5.744E-16	2.498E-15	100.000						
13	4.818E-16	2.095E-15	100.000						
14	3.341E-16	1.453E-15	100.000						
15	2.482E-16	1.079E-15	100.000						
16	2.028E-16	8.816E-16	100.000						
17	3.097E-17	1.346E-16	100.000						
18	1.606E-17	6.983E-17	100.000						
19	-3.120E-17	-1.357E-16	100.000						
20	-1.967E-16	-8.551E-16	100.000						
21	-2.977E-16	-1.294E-15	100.000						
22	-3.682E-16	-1.601E-15	100.000						
23	-5.662E-16	-2.462E-15	100.000						

Extraction Method: Principal Component Analysis (PCA).

Table 3b. Total Variance Explained of PCA for Figure 5b in liver.

Total Variance Explained

Component	Initial Eigenvalues			Extraction Sums of Squared Loadings			Rotation Sums of Squared Loadings		
	Total	% of Variance	Total	Total	% of Variance	Total	Total	% of Variance	Total
1	12.934	56.235	56.235	12.934	56.235	56.235	9.199	39.997	39.997
2	5.167	22.465	78.700	5.167	22.465	78.700	8.695	37.805	77.802
3	3.035	13.197	91.897	3.035	13.197	91.897	3.242	14.095	91.897
4	.714	3.104	95.001						
5	.499	2.171	97.172						
6	.197	.856	98.029						
7	.144	.627	98.656						
8	.126	.548	99.203						
9	.091	.396	99.599						
10	.061	.266	99.865						
11	.031	.135	100.000						
12	4.936E-16	2.146E-15	100.000						
13	3.898E-16	1.695E-15	100.000						
14	2.809E-16	1.221E-15	100.000						
15	1.566E-16	6.811E-16	100.000						
16	4.265E-17	1.854E-16	100.000						
17	-6.197E-17	-2.694E-16	100.000						
18	-1.045E-16	-4.543E-16	100.000						
19	-2.169E-16	-9.430E-16	100.000						
20	-3.495E-16	-1.520E-15	100.000						
21	-4.130E-16	-1.796E-15	100.000						
22	-5.629E-16	-2.447E-15	100.000						
23	-7.041E-16	-3.061E-15	100.000						

Extraction Method: Principal Component Analysis (PCA).

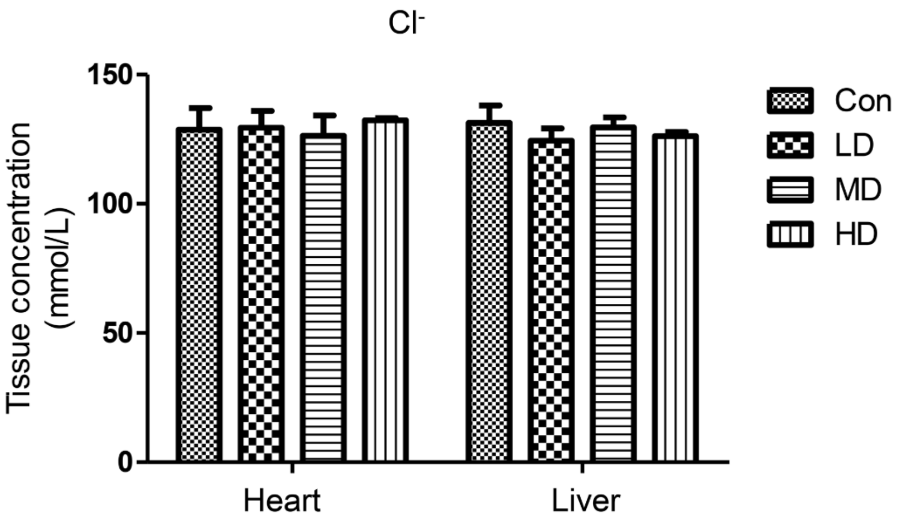

Fig. S1. The Cl^- contents in heart and liver.

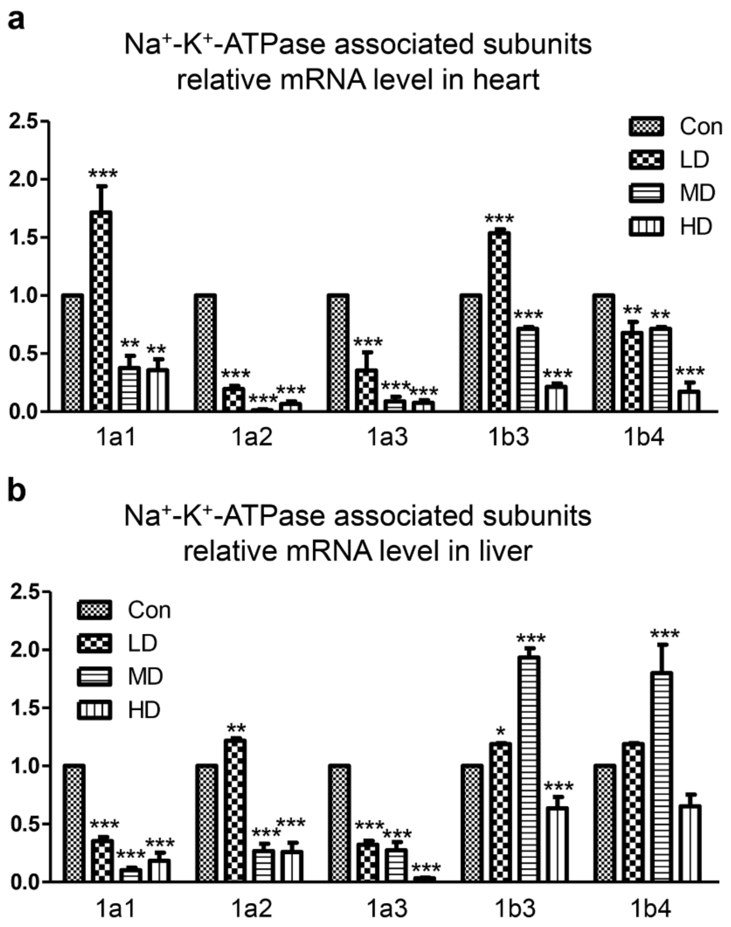

Fig. S2. The transcription of Na$^+$-K$^+$-ATPase associated subunits in heart and liver. (a) The transcription of Na$^+$-K$^+$-ATPase associated subunits in heart; (b) The transcription of Na$^+$-K$^+$-ATPase associated subunits in liver. Values were expressed as mean ± S.D.. Symbol for the significance of differences between the vehicle control and another: * $P < 0.05$, ** $P < 0.01$, *** $P < 0.001$.

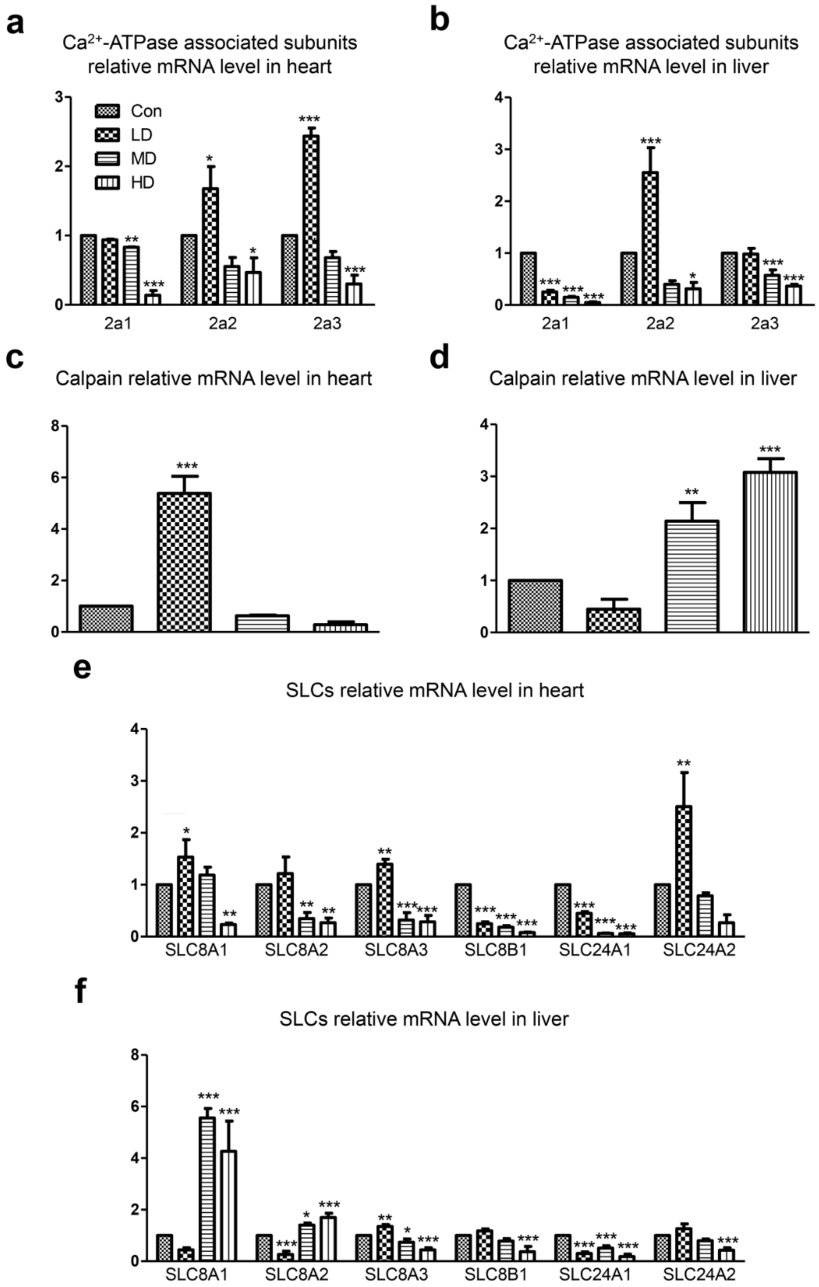

Fig. S3. The transcription of Ca^{2+}-ATPase associated subunits, calpain and SLCs in heart and liver. (a) The transcription of Ca^{2+}-ATPase associated subunits in heart; (b) T The transcription of Ca^{2+}-ATPase associated subunits in liver; (c) The transcription of calpain in heart; (d) T The transcription of calpain in liver; (e) The transcription of SLCs in heart; (f) T The transcription of SLCs in liver. Values were expressed as mean ± S.D.. Symbol for the significance of differences between the vehicle control and another: * $P < 0.05$, ** $P < 0.01$, *** $P < 0.001$.
